# Conference report of the 2024 Antimicrobial Resistance Meeting

**DOI:** 10.1038/s44259-024-00058-z

**Published:** 2024-11-29

**Authors:** Charlotte E. Chong, Thi Mui Pham, Megan E. Carey, Bryan A. Wee, Mona L. Taouk, Javier F. Favieres, Catrin E. Moore, Zoe A. Dyson, Cherry Lim, Connor L. Brown, Deborah Williamson, Lulla Opatowski, Kevin Outterson, Karyn M. Mukiri, Norelle L. Sherry, Sabiha Y. Essack, Sylvain Brisse, Yonatan H. Grad, Kate S. Baker

**Affiliations:** 1 Department of Genetics, University of Cambridge, Cambridge, United Kingdom; 2 Department of Immunology and Infectious Diseases Harvard T. H. Chan School of Public Health, Boston, MA USA; 3 Department of Infection Biology, Faculty of Infectious and Tropical Diseases, London School of Hygiene & Tropical Medicine, London, United Kingdom; 4 IAVI, Chelsea & Westminster Hospital London, London, United Kingdom; 5 The Roslin Institute, University of Edinburgh, Edinburgh, United Kingdom; 6 Department of Infectious Diseases, The University of Melbourne at the Peter Doherty Institute for Infection and Immunity, Melbourne, Victoria Australia; 7 Antimicrobial Resistance Unit (ARU) Animal Health Department, Faculty of Veterinary Medicine and VISAVET Health Surveillance Centre, Complutense University of Madrid, Madrid, Spain; 8 Centre for Neonatal and Paediatric Infection, City St. Georges, University of London, London, United Kingdom; 9 Wellcome Sanger Institute, Wellcome Genome Campus, Hinxton, United Kingdom; 10 Centre for Tropical Medicine and Global Health, Nuffield Department of Medicine, University of Oxford, Oxford, United Kingdom; 11 Mahidol Oxford Tropical Medicine Research Unit, Faculty of Tropical Medicine, Mahidol University, Bangkok, Thailand; 12 Virginia Tech, Department of Civil and Environmental Engineering, Blacksburg, VA 24061 USA; 13 Department of Medicine, University of St Andrews, St Andrews, United Kingdom; 14 Institut Pasteur, Université Paris Cité, Epidemiology and Modelling of Bacterial Escape to Antimicrobials (EMEA), 75015 Paris, France; 15 INSERM, Université Paris-Saclay, Université de Versailles St-Quentin-en-Yvelines, Team Echappement aux Anti-infectieux et Pharmacoépidémiologie U1018, CESP, 78000 Versailles, France; 16 Boston University School of Law, Boston, MA USA; 17 David Braley Centre for Antibiotic Discovery, McMaster University, Hamilton, ON Canada; 18 Michael G. DeGroote Institute for Infectious Disease Research, McMaster University, Hamilton, ON Canada; 19 Department of Biochemistry and Biomedical Sciences, McMaster University, Hamilton, ON Canada; 20 Microbiological Diagnostic Unit Public Health Laboratory, Department of Microbiology and Immunology, University of Melbourne at the Peter Doherty Institute for Infection and Immunity, Melbourne, VIC Australia; 21 Department of Infectious Diseases and Immunology, Austin Health, Heidelberg, VIC Australia; 22 Antimicrobial Research Unit, Department of Pharmacy, School of Health Sciences, University of KwaZulu-Natal, Durban, South Africa; 23 School of Pharmacy, University of Jordan, Amman, Jordan; 24 Institut Pasteur, Université Paris Cité, Biodiversity and Epidemiology of Bacterial Pathogens, Paris, France

**Keywords:** Biological techniques, Biotechnology, Computational biology and bioinformatics, Drug discovery, Evolution, Genetics, Immunology, Microbiology, Molecular biology, Diseases, Health care, Medical research, Pathogenesis, Risk factors

## Abstract

The Antimicrobial Resistance - Genomes, Big Data and Emerging Technologies Conference explored key topics including measuring the burden of AMR, global public health pathogen genomics infrastructure and surveillance, translation and implementation of genomics for AMR control, use of techniques such as wastewater surveillance, mathematical and statistical modelling, and Artificial Intelligence (AI) to aid understanding of AMR. This report describes research presented during plenary sessions and discussions, keynote presentations and posters.

## Introduction

Wellcome Connecting Science Learning and Training hosted the fourth iteration of the Antimicrobial Resistance - Genomes, Big Data and Emerging Technologies Conference on 13–15^th^ March 2024, in Hinxton, UK. The meeting brought together 183 in-person and 638 virtual attendees from 99 different countries with the aim of sharing research on a range of themes including, genomic surveillance, AI, machine learning, and wastewater surveillance addressing key questions in the field such as the burden of AMR.

Research results covering many aspects of AMR were reported in two keynote addresses and 20 oral and 90 poster presentations. In the opening keynote, Professor Kevin Outterson (CARB-X) presented the complex interplay of legal and social factors in combatting AMR, evaluating multifaceted approaches such as extending intellectual property patents, addressing market failures, funding early-stage research, and advocating for global governance to bridge the significant funding gap and ensure equitable access, innovation, and stewardship in addressing the problem. The second keynote, Professor Deborah Williamson highlighted the importance of genomics in early detection, diagnostic design, targeted treatments, and surveillance of STIs, alongside biomedical prevention strategies, as part of dedicated public health responses.

The following report provides a summary of the research presented in each of the sessions and poster presentations. Our report is intended to share the meeting content with the wider scientific community to encourage collaboration and advocacy.

## Measuring the burden of antimicrobial resistance

Antimicrobial resistance (AMR) is a long-standing global public health threat. Measuring the burden of AMR is crucial for guiding, prioritizing, and evaluating interventions and policy decisions. However, obtaining accurate estimates of the burden of AMR raises multifaceted challenges in public health and epidemiology^1^. The scarcity of high quality, patient-level microbiology data linked to clinical outcomes, particularly in lower- and middle- income countries (LMICs), hinders accurate global AMR burden estimations. Additionally, several conceptual and methodological challenges complicate the quantification of AMR burden^2,3^. The conceptual obstacles stem from the complexity of defining “burden” itself^1,4^. The burden of AMR can be categorized into two main dimensions: (1) health burden and (2) economic burden^3,5^. Common metrics for health burden are the excess length of hospital stay, disability-adjusted life years, years of life lost, and mortality rates. The conceptual complexity is compounded by the question of which counterfactuals are appropriate when determining the burden^1,6^. A widely used counterfactual is antibiotic-susceptible infection, and in this case the health impact of antibiotic-resistant infection is estimated by comparing health outcomes of patients with antibiotic-resistant infections with those that would be expected if all resistant infections had been replaced with otherwise equivalent antibiotic-susceptible infections. The implicit assumption is that interventions to reduce antibiotic-resistant infections would result in antibiotic-susceptible infections taking their place, reducing resistance but not affecting the total number of infections. An alternative approach is to use a no infection counterfactual. In this case, the health impact of antibiotic-resistant infection is estimated by comparing health outcomes in patients with antibiotic-resistant infections with those that would be expected had the infections not occurred. The assumption is that removing antibiotic-resistant infections would have no influence on the chance of acquiring antibiotic-susceptible infections. All current approaches depend on passively collected surveillance data or observational studies to inform counterfactual outcomes and estimates are vulnerable to potentially important biases, such as inadequate adjustment for key variables, measurement bias due to delays in microbiology testing that is common in LMICs^7^ and, for community-acquired infections, collider bias when observations are selected from hospital databases only and conditioning on hospitalisation introduces a non-causal association between resistance and patient outcomes^8^. Target trial emulation offers one solution for identifying potential biases and designing analytical approaches to minimise the biases^9^.

Numerous efforts have been made to measure the burden of AMR in countries worldwide^2,10–14^. The most comprehensive data on the global health burden of AMR were presented in the Global Research on Antimicrobial Resistance(GRAM) study^2^. Based on 23 pathogens, and 88 pathogen–drug combinations in 204 countries and territories in 2019, the authors estimated 1.27 million deaths attributed and 4.95 million deaths associated with AMR in 2019. The highest burden of AMR was in low-income settings; the highest death rates were estimated for sub-Saharan Africa and South Asia. These results relied on modelling, with small amounts of empirical data derived from tertiary hospitals, and thus come with uncertainties. The authors highlighted how the scarcity and inconsistency of data, especially in LMICs, exacerbated the challenges in estimating the burden of AMR. Other studies were similarly limited^10,12^. A systematic review and meta-analysis of inpatient bloodstream infections (BSIs) in LMICs reported that AMR was associated with a 58% increase in BSI mortality rates, doubled the odds of ICU admissions, extended hospital stay by one week, and increased direct medical costs by approximately $12,000 per case^15^.

In a recent prospective cohort study (Mortality from Bacterial Infections Resistant to Antibiotics (MBIRA)), performed in eight sub-Saharan African hospitals from 2020-2022, Enterobacterales BSIs greatly increased mortality, but third-generation cephalosporin-resistance status of the Enterobacterales was not associated with an increased mortality risk^10^. These results diverge from previous evidence and may be explained by healthcare seeking behaviour in LMIC settings, inadequate resources, and variation in access to antibiotics over time^4^.

In another prospective cohort study, where children admitted to three Kenyan hospitals were enroled and followed for six months after discharge, it was observed that *Escherichia coli* isolates acquired Extended-Spectrum Beta-Lactamase (ESBL) and carbapenem resistance before and during hospitalisation. Genomic analyses of samples found the presence of both sequence types associated with carriage and those known to cause invasive disease, indicating that carriage may precede the onset of actual disease.

In May 2015, a global action plan on AMR was adopted by the World Health Assembly to implement policies together with implementation plans to prevent, control, and monitor AMR^16^. While in 2023, 93% of the participating countries reported a National Action Plan, only 25% had been budgeted with monitoring in place^16^. The WHO Global Antimicrobial Resistance and Use Surveillance System (GLASS) was launched in 2015 to foster surveillance and inform strategies to contain AMR^14^. The most recent results from 87 countries showed that about half of *Klebsiella pneumoniae* BSIs were resistant to first-line antibiotics (third generation cephalosporins), leading to increased use of, and resistance to, second-line antimicrobials (carbapenems)^17^. Global AMR trend analyses pointed to a rise in resistant infections, notably in Gram-negative organisms, in healthcare facilities after 2020 compared to earlier years. Data heterogeneity and gaps hindered the accurate estimation of both health and economic AMR burden. Addressing the need for improved surveillance and data collection, the WHO is enhancing GLASS by focusing on good quality, more granular, individual-level and molecular data to characterize resistance mechanisms, In addition, the WHO introduced a two-pronged approach combining passive routine surveillance and periodic surveys^14^.

## Global public health pathogen genomics infrastructure

Real-world public health applications of genomic surveillance need to be supported by appropriate ecosystem infrastructure, such as effective networks and data visualisation platforms.

The African Centre of Excellence for Genomics of Infectious Diseases (ACEGID) was established to train the next generation of African genomic scientists, to generate an understanding of ongoing regional health threats, and enable proactive and effective outbreak response. ACEGID established genomic surveillance in Sierra Leone and Nigeria in response to the 2014 Ebola outbreak, during which time the group provided close to real-time whole genomic sequencing (WGS) and elucidated regional transmission^18^. During the 2015 Lassa fever (LASV) outbreak in Nigeria, the group sequenced the virus to investigate its origins^19^. Happi and colleagues developed rapid diagnostic tests (RDTs) for Ebola virus and Lassa virus using CRISPR-based tools^20^ and conducted a genome wide association study (GWAS) to identify host genetic signatures identified with fatal LASV outcomes^21^. The group conducted clinical metagenomic surveillance to describe local febrile aetiology^22^, which resulted in the discovery of novel rhabdoviruses circulating in West Africa^23^, and the identification of a novel clade of yellow fever virus in an outbreak in Nigeria in 2018^24^. ACEGID-led efforts also helped to form the basis for the SARS-CoV-2^25^ and Mpox virus genomic surveillance in Africa^26^.

A consortium of colleagues from the Centre for Genomic Pathogen Surveillance (CGPS) in Colombia, India, Nigeria, and the Philippines highlighted efforts to establish a roadmap for the implementation of WGS into existing AMR surveillance programmes^27^. This consortia works with data visualisation and analysis tools including Pathogenwatch (https://pathogen.watch)^28–30^, which facilitates rapid genomic data analysis on a single and multiple genome level and provides phylogenetic, temporal, geographic, and other custom context, and AMRwatch (https://amr.watch), which provides an overview of variant distribution and AMR data for World Health Organization (WHO) priority pathogens.

An interactive online dashboard, AMRnet, summarises WGS-derived data to inform public health action. AMRnet expands the TyphiNET data visualisation model (https://typhi.net)^31,32^ to include additional priority bacterial pathogens for which there is a clear use case (e.g., informing vaccine development or deployment). Data are summarised by country and year, offering a national view of the frequency of resistance to relevant drugs, which can be viewed as a global map. Users can generate plots of drug resistance frequencies over time and view the bacterial genotypes and AMR determinants underlying these trends. AMR surveillance dashboards offer a means of making complex genome data more broadly accessible but face several challenges requiring input from key pathogen stakeholders and layers of data infrastructure, including data analysis platforms to handle processing of raw data (e.g. Enterobase^33^ and Pathogenwatch^28–30^), appropriate typing schemes^34^, comprehensive understanding of correlations between AMR genotypes and phenotypes, and sufficient metadata to determine which genomes are suitable for pathogen surveillance. AMRnet developers are working with community stakeholders and other ongoing efforts to develop solutions for these complex challenges.

Efforts within Sciensano, the Belgian public health institute, to build an overarching public health information infrastructure that could integrate WGS, clinical, and epidemiological data into existing surveillance activities and capacity were also described. The be.Prepared (Belgian Preparedness Architecture for Infectious Diseases) programme features pathogen-specific analytic pipelines and an intuitive user interface. In practice, challenges including data interoperability, use of different tests over time, and lack of stakeholder commitment limited the success of this programme. It was suggested that top-down decision-making is critical when establishing a national surveillance program incorporating patient-level data. Discussion following this talk highlighted efforts led by the Public Health Alliance for Genomic Epidemiology (PH4AGE) to promote interoperability in public health bioinformatics, including the hAMRonization package, which parses the outputs of multiple AMR gene detection tools into a common data structure^35^.

## Genomic surveillance of priority pathogens

Genomic surveillance enhances our ability to detect, monitor and control infectious diseases. The monitoring of pathogens enables early detection of outbreaks, variants and further understanding of transmission dynamics, AMR, vaccine development and control strategy, all of which are vital to global public health.

Global sequencing initiatives such as the Global Pneumococcal Sequencing Project (GPS), aim to strengthen the worldwide genomic surveillance of *Streptococcus pneumoniae* (https://www.pneumogen.net/gps/). Genomic surveillance through the GPS has allowed researchers to assess how the global pneumococcal population has evolved in the face of the Pneumococcal Conjugate Vaccine and its impact on AMR from 2011-present. Through genomic studies of *S. pneumoniae* from surveys across multiple countries, it was found that the PCV, which targets up to 20 serotypes commonly AMR, does reduce AMR in pneumococci, but the magnitude of the reduction depends on the antibiotic-selective pressure and serotype replacement dynamics^36,37^. The vaccine’s introduction in France led to a decrease in overall cases but a surge in meningitis cases caused by serotypes not covered by the vaccine such as 24F^38,39^. Vaccine evading strains with AMR can drive the increase in AMR if there is antibiotic-selective pressure present. These strains, present in the GPSC10 lineage, are globally distributed, highly invasive and resistant^38,39^. Stewardship measures are crucial post-vaccination to manage AMR effectively, particularly in high-burden regions.

Similarly, other studies used pre- and post- vaccine surveillance data to assess the impact of the Typhoid Conjugate Vaccine (TCV). The Surveillance of Enteric fever in Asia Project (SEAP) which started in 2016, identified high incidence of disease, hospitalisation, and XDR emergence in *Salmonella Typhi*^40^. Following this, the WHO recommended TCV should be included in routine immunisation schedules of countries with a high disease burden. TCV was introduced in Sindh, Pakistan in late 2019. The enteric surveillance data from the pre-vaccine period obtained from SEAP^40^ in Karachi was compared to the ongoing post-vaccine data from the Impact of TCV after Routine Immunisation Program in Pakistan (ITRIPP) project. A significant reduction in culture-confirmed typhoid cases among different age groups was found following the introduction of TCV in both vaccine-eligible and non-eligible age groups. Additionally, no significant differences were found in overall case numbers of paratyphoid and AMR between *S. Typhi* isolates from pre and post vaccination isolates. The study underscores the importance of TCV and the need for continued efforts in routine immunisation to further mitigate enteric fever.

The monitoring of pathogens across countries and over time helps to describe transmission dynamics and the spread of AMR. Pathogens of concern, such as *Neisseria gonorrhoeae* may be routinely whole genome sequenced and characterised in public health laboratories. In Australia, the annual incidence of gonorrhoeae notifications has increased by 100% since 2013, with the rise in AMR potentially leading to untreatable infections. Genomic analyses of over 6000 *N. gonorrhoeae* isolates, representing over 25% of clinical cases during the study period, revealed a decline in genomic diversity and transmission during the COVID-19 pandemic and persistent transmission clusters that were associated with heterosexual networks and patterns of AMR.

## Translation and implementation of genomics for antimicrobial resistance control

Genomic technologies have enabled monitoring of the spread, persistence and abundance of AMR genes and mutations in bacterial populations. This has enabled research to inform treatment guidelines and development. Implementing genomic-based surveillance for AMR control is a multifaceted endeavour encompassing challenges in bioinformatics, global initiatives to bolster sequencing capacity, accreditation for use in clinical and public health microbiology (CPHM), and efforts to improve predictive accuracy. Many initiatives have been implemented to increase sequencing and bioinformatic capacities in public health laboratories globally. SeqAfrica, supported by UK Aid Fleming Fund, aims to bolster genomic sequencing and bioinformatic capacity in LMICs in Africa, procuring sequencing instruments and engaging health professionals through training modules. Over 30,000 bacterial and COVID-19 genomes from 22 African countries have been collected in over four years as part of this endeavour, with 73% of these shared publicly (https://antimicrobialresistance.dk/seqafrica.aspx). Similarly, EURGen-RefLabCap, supported by an EC/HaDEA Grant, aims to strengthen, and modernise European public health laboratories using WGS, including simulation exercises for AMR outbreak scenarios in priority healthcare associated pathogens such as *Salmonella enterica* and *Campylobacter jejuni* (https://www.eurgen-reflabcap.eu/). GenEpi-BioTrain aims to enhance pandemic response capacity, as well as AMR mitigation, by funding a training programme to support capacity building in genomic epidemiology and bioinformatic skills, underscoring the importance of overcoming implementation challenges for global health.

In Canada, co-ordinated sequencing efforts have shed light on the dynamics of AMR for major causes of bacteraemia in Alberta, a metropolitan area, between 2006 and 2022. A comprehensive BSI cohort comprising over 38,000 isolates and 12 species, shows despite a decline in hospital-onset bacteraemia across many species, the persistence of susceptible cases to first and second-line treatments underscores the need for tailored infection control strategies (unpublished). Furthermore, the distinct population structure observed in different species and the increase in multidrug resistance among Gram-negative bacteria highlight the importance of targeted interventions to mitigate the spread of AMR.

Improving the predictive accuracy of bioinformatic tools and the clinical applicability of data is paramount for the reliable implementation of genomics in AMR surveillance. For instance, the Resistance Gene Identifier is instrumental in this endeavour, using data from the Comprehensive Antibiotic Resistance Database (CARD) for both genomic and metagenomic annotation context. CARD offers a robust informatics framework, combining its Antibiotic Resistance Ontology with curated AMR gene (ARG) sequences and resistance-conferring mutations^41^. Its ontology-centric data encompasses a vast array of hand-curated information, including reference sequences, mutations, and AMR detection models. In turn, this data facilitates the annotation and interpretation of bacterial resistomes and improves the specificity and sensitivity of AMR prediction, ensuring more accurate identification of resistance determinants in bacterial genomes^41^. However, the curation of CARD’s bioinformatic models relies solely on bitscores (a measure of sequence similarity), which may potentially contribute to the underprediction of ARGs. Mitigation efforts, such as focussing on additional sequence similarity metrics and incorporating phylogeny into CARD curation practices, may decrease the prevalence of false-negative ARG predictions .

Accreditation by ISO is key for genomic AMR detection tools and pipelines to be routinely used in CPHM. To address this, abriTAMR, tailored for detecting AMR with command line capabilities and compatibility with most bacterial species, including some phenotypic inference was developed and validated to ISO standards^42^. In the tuberculosis (TB) context, a wish-list for mutational resistance detection, command line functionality, high accuracy, and ISO standard validation was pursued, leading to validated AMR detection from broth cultures, integrating genomic and phenotypic data^43^. Combining these efforts, in the case of carbapenemase-producing Enterobacterales (CPE), prospective genomic surveillance proved instrumental in untangling outbreaks of CPE in Victoria, Australia and implementing state-wide surveillance programs, helping to stabilize notifications^44^. Implementation of genomics in hospital infection control marked a paradigm shift, requiring effective communication between teams, understanding clinical interpretations alongside bioinformatics goals, and tailoring reports to meet the needs of infection control teams, ultimately emphasizing the importance of interdisciplinary collaboration and effective communication for successful implementation.

## Wastewater surveillance

Wastewater surveillance of AMR is an increasing focus of research that could fill a critical data gap by capturing community-level information about the prevalence of resistance, independent of care-seeking behaviour and availability of clinical infrastructure^45–47^. Historical examples where wastewater surveillance would have been especially useful were discussed, particularly in cases of rapid global spread of antibiotic resistance genes. Examples included the international spread of *bla*_NDM-1_, reported in Sweden in 2008^48^ and the first identification of *mcr-1* in the US in 2016^49^. The potential for wastewater surveillance in LMICs, and in tracing the emergence of future drug resistance genes, was highlighted.

A study from researchers at Virginia Tech sampled both the influent and treated wastewater of a conventional wastewater treatment plant (WWTP) twice weekly for one year. Deep metagenomic sequencing showed that resistance profiles in sewage resembled those of clinical bacteria, and that there was a lagging association with antibiotic usage in autochthonous members of the sewer system. Consistent with well-established trends^50,51^, it was found that taxa not commonly found in human stool harboured AMR genes and displayed associations with antibiotic resistance. It was hypothesised that the exposure to antibiotics in wastewater pipes selects for resistant environmental strains. A related study using overlapping datasets^52^ found differences in baseline resistance between two neighbouring wastewater treatment plants that was correlated with overall community antibiotic use.

Larger projects have sought to monitor infectious diseases in broader areas, including the CDC’s National Wastewater Surveillance System (NWSS), which commenced during the COVID-19 pandemic^53^. The Chief Scientific Officer of NWSS, Amy Kirby, detailed the weekly sampling of NWSS from more than 1500 WWTPs covering about 40% of the U.S. Current efforts at NWSS are centred on viral dd-PCR with some viral variant sequencing^54^. These data can be of great use to health authorities, by informing decisions such as the timing and location of testing and vaccination sites. Limitations were highlighted, such as the number of households not connected to the sewage system, variation between systems and noise data like population movement, rainfall, and industrial spill over. While NWSS is currently screening only for the detection of SARS-CoV-2 and the Mpox virus, it is preparing to survey AMR genes. Challenges remain, such as how to interpret the results of such monitoring, and in establishing concentrations of antibiotic resistance genes that should cause concern. In addition, the specific technology is still being explored, including dd-PCR or metagenomics with the latter being an especially important issue given the differences in both procedures’ implementation and output.

Metagenomic analyses can search for hundreds of reference genes, detect new ones, and give information on the genomic contexts of those found^55^. However, protocols are not standardised, their analyses are costly and time-consuming, and a high depth is needed to uncover AMR genes. High Throughput Quantitative PCR (HT-qPCR), on the other hand, yields results more quickly but is restricted to targeted genes^56^. To assess their capabilities, a recent study compared both methods on the same wastewater samples. A higher sensitivity was demonstrated for HT-qPCR, although several AMR genes were detected solely by metagenomic analyses. These results highlight the importance of previous knowledge about the samples for informed target gene choice.

## AI, ML and big data tools

Mathematical and statistical models are helpful in identifying patterns in large datasets to understand the drivers of AMR. To explore the role of human interactions, transmission network modelling has shown that for some organisms such as ESBL producing *K. pneumoniae*, contact networks between people can be used to explain the transmission of the pathogen. These models also showed that transmissibility could be higher for some resistant lineages, which may require targeted strategies to contain outbreaks^57,58^. However, human interactions were not found to be a major driver for other species such as ESBL producing *E. coli*. To further explore the heterogeneity in global rates of resistance as captured in large databases such as the industry-funded Atlas database, models were used to investigate how resistance can be related to country-specific factors (https://www.atlas-surveillance.com/). These findings suggest that not all resistances in drug-bacteria combinations were linked solely to antibiotic consumption in humans and are rather associated with a combination of factors^59^. This suggests that intervention and control measures should be tailored to specific drug- bacteria combinations. Further modelling of the same data, especially in ESBL producing *E. coli* suggests that heterogeneity of AMR transmission and spread exists at the global scale, and the importance of globally representative, country-specific surveillance data from healthy humans will be key to refine our understanding of the problem and inform better on the expected impact of interventions. Looking ahead, these models can also be used to explore scenarios such as the emergence of super-bugs and other multidrug resistant bacteria at the country level and the global scale.

Machine learning approaches were also employed to make predictions from complex datasets. For example, the main cause of treatment failure in humans after antibiotic treatment is due to the emergence of antibiotic resistance. A study in urinary tract infections used machine learning methods to predict the likelihood of reacquiring a resistant infection from patients’ medical histories, demographics, and past antibiotic prescriptions. This algorithm can be used to support physicians’ decision-making when deciding on antibiotic prescriptions to minimise recurring infections and the risk of acquiring resistance^60^.

The U.S. Pharmacopeia (USP) also demonstrated the use of machine learning-based analytics on a large proprietary supply chain dataset called the Medicine Supply Map to monitor the stock levels and quality of antibiotics to ensure sufficient access to antibiotics where they are needed (https://www.usp.org/supply-chain/medicine-supply-map). Machine learning was also used to give context to transmission pathways of antimicrobial resistance genes based on how they are reported in the literature.

Large genomic datasets are also being used to understand the drivers of resistance with statistical approaches and novel bioinformatic tools. Shotgun metagenome sequences from the human gut microbiomes were used to identify the environmental and demographic factors associated with antibiotic resistance gene load. This was described using a Finnish cohort, which will be expanded by investigating using publicly available metagenomic datasets from global human gut microbiome studies^61^. There was also ongoing work to define how transmission vehicles of AMR, such as plasmids, could be better understood in these large sequencing datasets and the application of Bayesian methods to improve the genome-wide association of sequences with phenotypes of interest, such as resistance.

## Conclusions

The summary of research presented at the 2024 Antimicrobial Resistance- Genomes, Big Data and Emerging Technologies Conference, as illustrated here, provides insight into the global research currently taking place to evaluate the burden, drivers and ways to combat of AMR.

The conference highlighted the challenges in quantifying AMR and the importance of working backwards from the goal of reducing the burden of AMR. It brought together a community focused on sharing expertise across pathogen silos to continue to build the toolbox of interventions for AMR, including vaccines, new antimicrobials, hygiene initiatives, stewardship, infection prevention and control, diagnostics, and infection models to improve understanding.

## Supplementary information

Supplementary_conferenceAgenda

## Data Availability

No datasets were generated or analysed during the current study.
