# Supplementary material for: Conference report of the 2024 Antimicrobial Resistance Meeting
Source: NPJ Antimicrob Resist. 2024 Nov 29;2:43. doi: 10.1038/s44259-024-00058-z (PMC11721068; doi:10.1038/s44259-024-00058-z)
Supplement: Supplementary file 1 — Supplementary_conferenceAgenda [file 44259_2024_58_MOESM1_ESM.pdf]

Antimicrobial Resistance - Genomes, Big Data and Emerging Technologies

13-15 March 2024

Hybrid Conference Programme

Start (GMT)	Finish (GMT)	Presenter details
**Wednesday 13 March 2024**		
**12:00**	**12:50**	**Registration, lunch and networking**
12:35	*12:50*	*Briefing for Keynote & Session 1 speakers, microphone runners, chair, moderator & committee - Auditorium*
**12:50**	**13:00**	**Welcome**
		Scientific Programme Committee:<br>*Kate Baker, University of Cambridge, UK*<br>*Sylvain Brisse, Institut Pasteur, France*<br>*Sabiha Essack, University of KwaZulu-Natal, South Africa*<br>*Yonatan Grad, Harvard University, USA*
**13:00**	**14:00**	**Keynote 1**
		Chair: Yonatan Grad, Harvard University, USA<br>*Moderator: Kate Baker, University of Cambridge, UK*
13:00	14:00	Legal and Social Ecologies of Resistance<br>*Kevin Outterson, Boston University, USA*
14:00	14:05	Comfort break
**14:05**	**15:45**	**Session 1: Measuring the Burden of AMR**
		Chair: Stephanie Wai-U Lo, Wellcome Sanger Institute, UK<br>*Moderator: Kate Baker, University of Cambridge, UK*
14:05	14:35	Measuring the burden of AMR and beyond<br>*Catrin Moore, St. George's, University of London, UK*
14:35	15:05	From Advocacy to Reliable Estimates: Unpacking the Global Burden of Antimicrobial Resistance<br>*Silvia Bertagnolio, World Health Organization, Switzerland*
15:05	15:20	Acquisition and persistence of Extended-spectrum beta-lactamase (ESBL) and Carbapenem resistant (CRE) Escherichia coli carriage in hospitalized Kenyan children<br>*Caroline Chepngeno Tigoi Kipngeno, KEMRI/Wellcome Trust Research Programme, Kenya*
15:20	15:35	Rethinking how to quantify and interpret health impact of antimicrobial resistant infections in resource-limited settings<br>*Cherry Lim, University of Oxford, UK*
15:35	15:45	Session discussion
15:45	16:20	Refreshment break and networking

16:05	*16:20*	*Briefing for Session 2 speakers, microphone runners, chair & moderator - Auditorium*
**16:20**	**18:00**	**Session 2: Global public health pathogen genomics infrastructure**
		Chair: Sylvain Brisse, Institut Pasteur, France<br>*Moderator: Yonatan Grad, Harvard University, USA*
16:20	16:50	Genomic Surveillance and Characterization of Microbial Threats Facilitates Early Detection and Containment of Disease Outbreaks in West Africa<br>*Christian Happi, Redeemer's University, Nigeria*
16:50	17:20	Maximising value of genomic and enabling data for surveillance of AMR: Bottom-up and top-down approaches<br>*David Aanensen, Oxford University, UK*
17:20	17:35	AMRnet: An online dashboard for global surveillance of AMR priority pathogens<br>*Zoe A. Dyson, London School of Hygiene and Tropical Medicine, UK*
17:35	17:50	The (overly?) ambitious plan to integrate genomics and patient data in the surveillance of infectious disease and AMR in Belgium: Experiences from the be.Prepared Project<br>*Pieter-Jan Ceyssens, Sciensano, Belgium*
17:50	18:00	Session discussion
**18:00**	**18:45**	**Poster pitch talks for odd number posters**
**18:45**	**19:55**	**Poster session 1 - odd number posters with drinks reception**
19:55	21:55	Dinner
19:55		Bar open (card payments only)

Thursday 14 March 2024

07:30	09:00	Breakfast
09:15	*09:30*	*Briefing for Session 3 speakers, microphone runners, chair & moderator - Auditorium*
**09:30**	**11:10**	**Session 3: Genomic surveillance of priority pathogens**
		Chair: Sylvain Brisse, Institut Pasteur, France<br>*Moderator: Kate Baker, University of Cambridge, UK*
09:30	10:00	Impact of typhoid conjugate vaccine<br>*Farah Naz Qamar, Aga Khan University, Pakistan*
10:00	10:30	The impact of Pneumococcal Conjugate Vaccines on Antimicrobial Resistance in Pneumococcal Diseases<br>*Stephanie Wai-U Lo, Wellcome Sanger Institute, UK*
10:30	10:45	Plasmids carrying antibiotic resistance genes are special: they are more mobile and they evolve faster<br>*Charles Coluzzi, Institut Pasteur, France*
10:45	11:00	Genomic insights into the longitudinal transmission of Neisseria gonorrhoeae<br>*Mona Taouk, University of Melbourne, Australia*
11:00	11:10	Session discussion
11:10	11:45	Refreshment break and networking
11:30	*11:45*	*Briefing for Session 4 speakers, microphone runners, chair & moderator - Auditorium*
**11:45**	**13:25**	**Session 4: Translation and implementation of genomics for AMR control**
		Chair: Kate Baker, University of Cambridge, UK<br>*Moderator: Sylvain Brisse, Institut Pasteur, France*
11:45	12:15	Adventures in AMR genomics at the clinical and public health microbiology coalface<br>*Norelle Sherry, University of Melbourne, Australia*
12:15	12:45	Leaving Pasteur and Fleming behind by implementing genomic-based surveillance for AMR<br>*Rene Hendriksen, Technical University of Denmark, Denmark*
12:45	13:00	Increasing the Predictive Accuracy of the Resistance Gene Identifier by Abandoning Sole Reliance on Bitscore<br>*Karyn Mukiri, McMaster University, Canada*
13:00	13:15	Genomic epidemiology of strain and antibiotic resistance dynamics for four major causes of bacteremia in a Canadian metropolitan area, 2006-2022<br>*Thi Mui Pham, Harvard T.H. Chan School of Public Health, USA*
13:15	13:25	Session discussion
13:25	14:45	Lunch and networking
14:30	*14:45*	*Briefing for Session 5 speakers, microphone runners, chair & moderator - Auditorium*

**14:45**	**16:25**	**Session 5: Wastewater surveillance**
		Chair: Yonatan Grad, Harvard University, USA<br>*Moderator: Stephanie Wai-U Lo, Wellcome Sanger Institute, UK*
14:45	15:15	Diving into the sewers to track antimicrobial resistance<br>*Amy Kirby, National Wastewater Surveillance System, USA*
15:15	15:45	The utility of wastewater detection of antimicrobial resistance for public health and health system response<br>*Guy Palmer, Washington State University, USA*
15:45	16:00	Resistance in sewage-associated microbiota mirror patterns of clinical bacteria and reflect a variable lagging association with antibiotic usage<br>*Connor Brown, Virginia Tech, USA*
16:00	16:15	High Throughout qPCR is more sensitive than Metagenomics for wastewater AMR surveillance<br>*Nicola Coyle, Cefas, UK*
16:15	16:25	Session discussion
16:25	16:55	Refreshment break and networking
**16:55**	**17:40**	**Poster pitch talks for odd number posters**
**17:40**	**18:50**	**Poster session 2 - even number posters with drinks reception**
18:50	20:50	Dinner
18:50		Bar open (card payments only)

Friday 15 March 2024

07:30	09:00	Breakfast
09:15	*09:30*	*Briefing for Session 6 speakers, microphone runners, chair & moderator - Auditorium*
**09:30**		**Session 6: AI, ML, and Big Data Tools**
		Chair: Yonatan Grad, Harvard University, USA<br>*Moderator: Kate Baker, University of Cambridge, UK*
09:30	10:00	What can models tell us about the dynamics of AMR from large-scale data?<br>*Lulla Opatowski, Institut Pasteur, France*
10:00	10:30	Predicting Antibiotic Resistance<br>*Roy Kishony, Technion, Israel*
10:30	10:45	From Hindsight to Foresight: Safeguarding the Antibiotic Supply Chain - VIRTUAL<br>*Chaitanya Koduri, US Pharmacopeia, USA*
10:45	11:00	From Archives to Insights: Antimicrobial Resistance Analysis in Global-Scale Metagenomic Datasets<br>*Shivang Bhanushali, University of Turku, Finland*
11:00	11:10	Session discussion
11:10	11:45	Refreshment break and networking
11:30	*11:45*	*Briefing for Keynote, microphone runners, chair, moderator & committee - Auditorium*
**11:45**	**12:45**	**Keynote 2**
		Chair: Kate Baker, University of Cambridge, UK<br>*Moderator: Sylvain Brisse, Institut Pasteur, France*
11:45	12:45	Applying novel diagnostic approaches to the prevention and control of AMR<br>*Deborah Williamson, UK Health Security Agency, UK and University of Melbourne, Australia*
**12:45**	**13:00**	**Closing remarks and prize presentation**
		Scientific Programme Committee:<br>*Kate Baker, University of Cambridge, UK*<br>*Sylvain Brisse, Institut Pasteur, France*<br>*Sabiha Essack, University of KwaZulu-Natal, South Africa*<br>*Yonatan Grad, Harvard University, USA*
13:00	13:50	Lunch and departures
13:50		Coach departures for Stansted and Heathrow airports
14:00		Coach departures for Cambridge train station and city centre
